# High On-Treatment Platelet Reactivity as a Tool for Risk Stratification in STEMI Patients

**DOI:** 10.3390/jcm14176026

**Published:** 2025-08-26

**Authors:** Aleksandra Karczmarska-Wódzka, Patrycja Wszelaki, Szymon Szymoniuk, Krzysztof Pstrągowski, Joanna Sikora

**Affiliations:** 1 Research and Education Unit for Experimental Biotechnology, Department of Transplantology and General Surgery, Faculty of Medicine, Collegium Medicum in Bydgoszcz, Nicolaus Copernicus University in Toruń, 85-094 Bydgoszcz, Poland; akar@cm.umk.pl (A.K.-W.); wszelakipatrycja@cm.umk.pl (P.W.); szymon.szymoniuk@cm.umk.pl (S.S.); 2 Department of Cardiology and Internal Medicine, Antoni Jurasz University Hospital No. 1 in Bydgoszcz, 85-094 Bydgoszcz, Poland; pstragowski.krzysztof@gmail.com

**Keywords:** myocardial infarction, STEMI, platelets, antiplatelet drugs

## Abstract

**Background/Objectives:** In the last decade, several studies revealed individual response variability to different antiplatelet agents, and patients who have no response to these drugs are considered poor responders. Some studies explored platelet function during antiplatelet treatment to identify those patients with “high on-treatment platelet reactivity” (HPR), which exposes them to increased risk of major adverse cardiovascular events (MACE). **Methods:** We conducted a study with patients with ST-elevation myocardial infarction (STEMI) treated with dual antiplatelet therapy (DAPT) with ticagrelor and aspirin, including long-term follow-up after 5 years. We used thromboelastography, the total thrombus formation analysis system, and vasodilator-stimulated phosphoprotein phosphorylation assay (VASP) to analyze HPR with different methods; selected laboratory parameters were measured during hospitalization to check significant correlations. **Results:** We identified STEMI patients treated with DAPT with HPR as a risk group for MACE in a 5-year follow-up. Additionally, we have shown that HPR is associated with atherosclerosis by analyzing lipid profile parameters. **Conclusions:** High on-treatment platelet reactivity (HPR) increases the risk of major adverse cardiovascular events in the long term, especially with elevated C-reactive protein or an atherogenic lipid profile. Standardizing HPR assessment is crucial for optimizing individualized antiplatelet therapy and improving patient outcomes post-STEMI.

## 1. Introduction

Platelets were discovered by the Italian pathologist Giulio Bizzozero in 1882. He observed them microscopically in living animals’ circulating blood and blood taken from blood vessels. He also described in detail the function of platelets under flow conditions, the relationship between platelet adhesion and aggregation, and the subsequent formation and deposition of fibrin [1]. The basic features of platelet physiology began to be characterized at the molecular level by a small group of investigators from the late 1940s to the mid-1960s [2]. These studies included investigations on serotonin and platelet vasoconstriction, the release reaction, and the effects of thrombin and collagen on platelet aggregation. Eventually, it was hypothesized that erythrocytes contain a factor that could activate platelets [3]. This was later identified as adenosine diphosphate (ADP) [4]. Further studies demonstrated the role of ADP-induced platelet activation/aggregation in coronary artery thrombosis, and the era of antiplatelet drugs began and revolutionized many areas of medicine.

Currently, there are several groups of antiplatelet drugs with different mechanisms of action. Along with these new drugs, methods have been developed to monitor platelet activity. Many studies indicate that there are patients who, despite receiving therapy, present high on-treatment platelet reactivity (HPR) and maintain high platelet activity. One of the first reports of HPR was a study by Gurbel et al. [5]. Their research showed that patients with the highest baseline platelet reactivity remained the least protected after treatment. This phenomenon of the predictive value of baseline reactivity was later confirmed in many studies as a risk factor for ischemic events. These results suggested that the standard dose of clopidogrel is insufficient in some patients, which created the basis for further studies on intensification of therapy (e.g., higher doses, new-generation inhibitors such as prasugrel or ticagrelor). Multiple studies have demonstrated a relationship between HPR measured by multiple platelet assays and adverse clinical ischemic events. However, due to a lack of consensus on the optimal methods to assess HPR and the cutoff values, routine platelet reactivity monitoring has not been widely implemented in clinical practice or recommended in the proper guidelines [6].

Currently available evidence supports the concept of a threshold for on-treatment platelet reactivity that may be used to stratify patient risk for ischemic/thrombotic events following percutaneous coronary intervention (PCI), including stent thrombosis. Despite some recommendations in the literature, it is not easy to provide exact values that define HPR using different methods. This study aimed to assess HPR in ST-evaluation myocardial infarction patients treated with one of the more potent ADP inhibitors—ticagrelor. HPR was evaluated using several methods, including thromboelastography (TEG), the total thrombus formation system (TTAS), and VASP. Additionally, whether the identification of HPR affects the risk stratification of patients during a 5-year follow-up was assessed.

## 2. Materials and Methods

### 2.1. Study Group

The trial was conducted according to the Declaration of Helsinki and Good Clinical Practice guidelines. The protocol of the study was approved by the Ethics Committee of Nicolaus Copernicus University in Toruń, Collegium Medicum in Bydgoszcz (approval number KB 207/2015). Each patient provided written informed consent to participate in the study before enrollment. Briefly, the inclusion criteria consisted of a diagnosis of acute coronary syndromes (ACS) and the qualification of patients for invasive diagnostics: in the case of STEMI, up to 6 h from the onset of symptoms, and in the case of NSTEMI patients, from the very high- and high-risk groups. The exclusion criteria consisted of current treatment with any antiplatelet or anticoagulant agents, chronic therapy with low-molecular-weight heparin, any previous invasive or structural procedures, active bleeding, history of gastrointestinal bleeding, a history of coagulation disorders, any kidney or hepatic disorder, respiratory failure, history of severe chronic heart failure, history of major surgery, or concomitant therapy with potent CYP3A inhibitors or inducers. Any unexpected complications during or after PCI or hospitalization, including but not limited to unstable hemodynamic state or cardiogenic shock, life-threatening cardiac arrhythmias or cardiac arrest, mechanical complications of MI, and stent thrombosis, were excluded from the study and not included in the final analysis, as shown in the flowchart.

### 2.2. Study Outcomes

The primary endpoint of this study was the percentage of patients with high platelet reactivity (HPR) at 24 h following the administration of DAPT. The platelet reactivity was assessed with VASP, TTAS, and TEG, with the area under the platelet reactivity index (PRI) assessed by VASP assay, the area under the aggregation curve (T-TAS_AUC10) assessed by TTAS, and the maximum amplitude (MA) and percentage of inhibition platelet mapping ADP (MAP_InhADP) by TEG. The secondary endpoint was the incidence of major adverse cardiovascular events (MACE) during a 5-year follow-up period. 

### 2.3. Platelet Reactivity Assessment

Platelet function and clot formation were assessed using three independent methods. We used the VASP assay (Biocytex, Inc., Marseille, France), T-TAS (Fujimori Kogyo Co., Tokyo, Japan), and TEG 5000 (Haemonetics Corp., Braintree, MA, USA). The PL-chip by T-TAS was used to measure platelet thrombus formation under physiological conditions on a collagen-coated analytical pathway consisting of microcapillary channels. The thromboelastography assessment included two distinct tests: the kaolin-activated TEG, which evaluates an intrinsic pathway activated assay, and TEG PlateletMapping, which consists of a thrombin-generated tracing and platelet receptor-specific tracings to identify the level of platelet inhibition and aggregation. The following thresholds defined high platelet reactivity: PRI > 30%, T-TAS_AUC10 > 50 AUC, MA > 73 mm, MAP_InhADP > 70%, assessed with VASP, TTAS, and TEG, respectively. Because the patients were treated with the strong antiplatelet therapy ticagrelor with aspirin, we decided to lower the reference values most commonly used for the VASP and TTAS, PRI > 30% instead of 50% [7], T-TAS_AUC10 > 50 AUC instead of 260 AUC [8], respectively. Our observations and experience show that reference values should be differentiated due to the varying potency of different antiplatelet drugs. Many authors also emphasize this point, especially when comparing clopidogrel/ticagrelor therapy [9,10,11].

### 2.4. Statistical Analysis

Pharmacodynamic, diagnostic, biochemical, and coagulation numerical variables are presented as the median (inter-quartile range (IQR)), with additional descriptive information provided in brackets where appropriate. Categorical variables are presented as percentages. The Shapiro–Wilk test was used to assess the normality of distribution for numerical continuous variables. Depending on the distribution, group comparisons for numerical continuous variables were performed using the independent samples *t*-test or the Mann–Whitney U test. Group comparisons were conducted using the Chi-square test, as appropriate for categorical variables. Spearman’s rank correlation coefficients were calculated to explore relationships between variables where suitable. Patient survival from the time of discharge was analyzed using Kaplan–Meier curves. A *p*-value of less than 0.05 was considered statistically significant for all analyses. All statistical analyses were performed using SPSS Statistics, version 29.0.0.0.

## 3. Results

### 3.1. Baseline Characteristics and In-Hospital Events

Out of 102 patients diagnosed with ACS, who were admitted to the Department of Cardiology and Internal Medicine, 82 were included in the study (36 STEMI, 22 non-ST-elevation myocardial infarction (NSTEMI), and 18 unstable angina (UA)), according to the inclusion/exclusion criteria, detailed in the Materials and Methods. After admission to the study center (Cardiology Clinic, Dr A. Jurasz University Hospital no 1, Bydgoszcz, Poland) and confirmation of the initial diagnosis of ACS, all patients received a 300 mg loading dose (LD) of aspirin and 180 mg LD of ticagrelor. Subsequently, all patients underwent a coronary angiography assessment followed by percutaneous coronary intervention (PCI), if necessary. Six patients were excluded from the study during hospitalization (one cardiogenic shock during coronary angiography, two qualified for coronary artery bypass graft (CABG), two necessitated abciximab administration, and one death). Initially, all patients with ACS were included in the study, each receiving the same antiplatelet therapy. Because not all PCI is urgent, the next blood draw was scheduled 24 h after the DAPT LD. After analysis of the results, HPR was identified in all patients, but the highest percentage occurred in STEMI patients. Therefore, the decision was made to include STEMI patients for further analysis.

**Figure 1:**
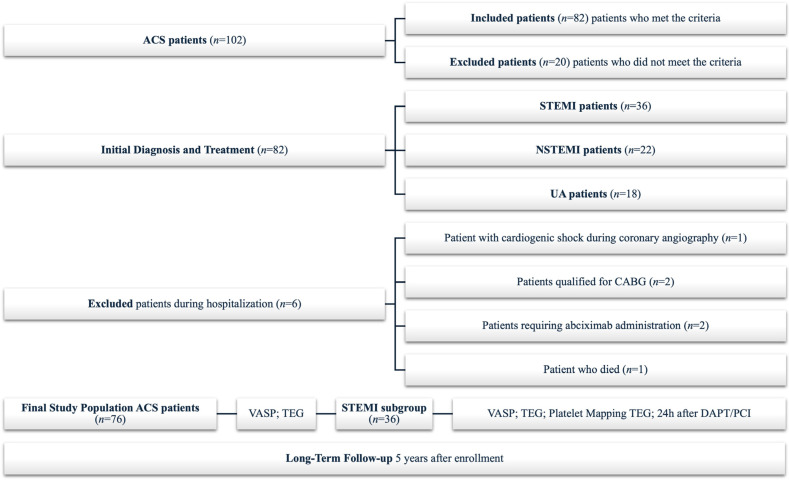
Trial flowchart. ACS: acute coronary syndromes; CABG: coronary artery bypass grafting; VASP: vasodilator-stimulated phosphoprotein phosphorylation assay; TEG: thromboelastography; STEMI: ST-elevation myocardial infarction; DAPT/PCI: dual antiplatelet therapy/percutaneous coronary intervention.

A subgroup of patients diagnosed with STEMI was identified for further analysis to apply additional methods that may identify patients with HPR. Five years after enrolment in the study, we followed up patients to examine the long-term effects related to MACE, defined as any of the following events: myocardial infarction, heart failure, stroke, ischemic heart disease, thrombosis, or death from cardiovascular causes. The analysis included the first occurrence of any of these events. A flowchart of the study is presented in Figure 1.

### 3.2. High On-Treatment Platelet Reactivity in ACS

**Figure 2:**
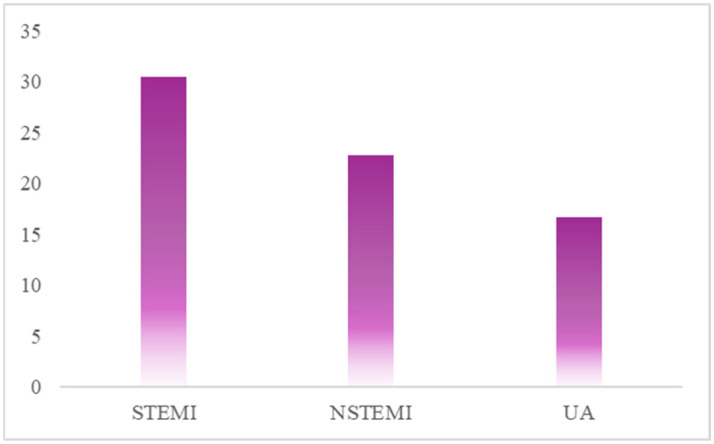
Occurrence of HPR determined by maximum amplitude measured by thromboelastography in patients with acute coronary syndromes. STEMI: ST-elevation myocardial infarction; NSTEMI: non-ST-elevation myocardial infarction; UA: unstable angina.

The chart in Figure 2 displays the percentage of patients identified with HPR using the MA parameter from TEG. The data are presented in the separate patient groups: STEMI, NSTEMI, and UA. The results indicate that the STEMI patient group presents the highest rate of HPR, with an incidence of over 30%. This value is significantly higher when compared to the NSTEMI group, where HPR prevalence is approximately 23%, and the UA group, where it is approximately 16% (Figure 2).

### 3.3. High On-Treatment Platelet Reactivity in STEMI

**Figure 3:**
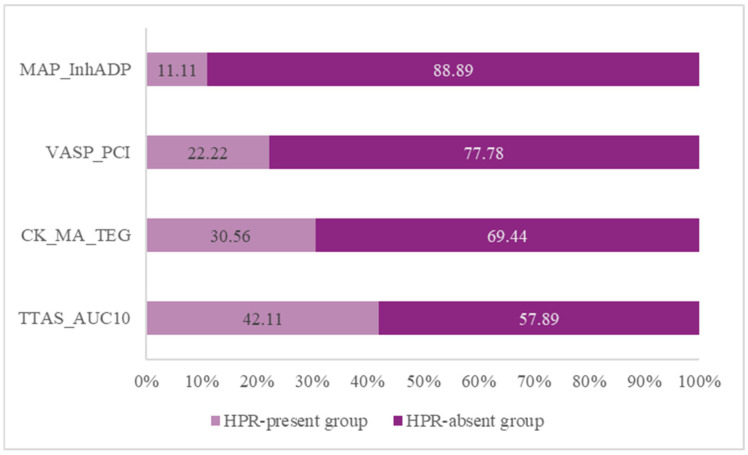
Detection rate of HPR across different methods. MAP: platelet mapping; InhADP: percentage of inhibition platelet mapping ADP; ADP: adenosine diphosphate; VASP: vasodilator-stimulated phosphoprotein phosphorylation assay; PCI: percutaneous coronary intervention; CK: kaolin test; MA: maximum amplitude; TEG: thromboelastography; TTAS: total thrombus formation analysis system; AUC10: area under the pressure–time curve for 10 min, PL chip for T-TAS; HPR: high on-treatment platelet reactivity.

For each method, Figure 3 shows the percentage of patients who belong to the HPR-present group versus the HPR-absent group. The number of patients with HPR is different for each method. The T-TAS_AUC10 identified the highest percentage of HPR-present patients at 42.11% (Figure 3). The HPR presence is highly dependent on the method used.

Table 1 shows the patients identified as HPR-present by each method. For instance, 15.79% of all patients were identified as having HPR by the T-TAS_AUC10 and CK_MA_TEG methods, indicating the highest level of similarity between any two methods. In contrast, there was a complete lack of HPR agreement between the CK_MA_TEG and MAP_InhADP methods, although each of these tests is theoretically related to platelet reactivity.

### 3.4. HPR-Present vs. HPR-Absent

Since not every method identified patients with HPR in the same way, for analysis, we created a group that included patients in whom HPR was detected by at least one method. The basic characteristics of the studied subgroup and the division into groups are presented in Table 2.

### 3.5. Kaplan–Meier Survival Analysis

**Figure 4:**
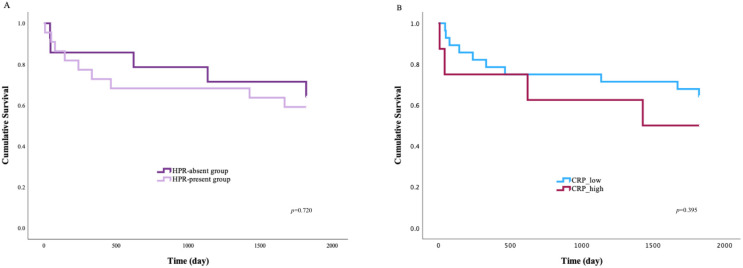
Kaplan–Meier survival curves according to the HPR group (**A**) and CRP levels (**B**), respectively. HPR: high on-treatment platelet reactivity; CRP: C-reactive protein; *p*-values obtained from the log-rank test.

**Figure 5:**
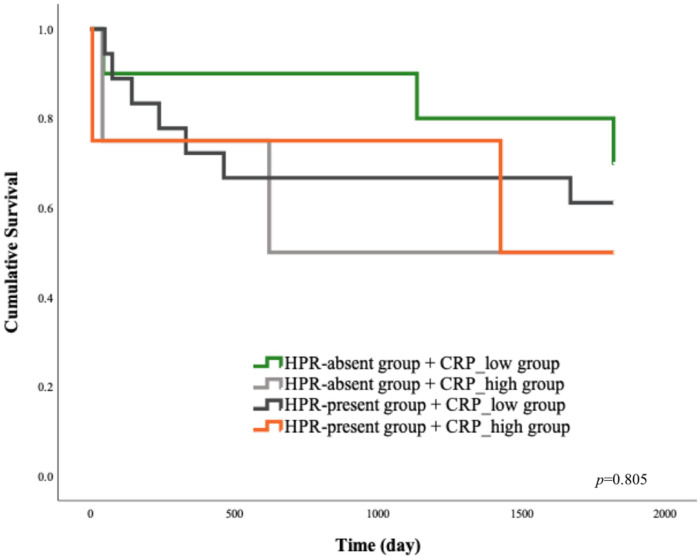
Synergistic prognostic value of HPR and CRP for risk stratification following STEMI. HPR: high on-treatment platelet reactivity; CRP: C-reactive protein; *p*-values obtained from the log-rank test.

Figure 4A presents the Kaplan–Meier survival curves comparing patients from the HPR-absent and HPR-present groups, where HPR was defined as being detected by one or more methods. The survival curve for the HPR-present group trends consistently below the curve for the HPR-absent group, which suggests a worse prognosis and a higher risk of MACE in patients with HPR. Additionally, the Kaplan–Meier graph compares the survival functions of patients categorized by low and high C-reactive protein (CRP) levels (Figure 4B). The survival curve for the CRP_high group is positioned below that of the CRP_low group, indicating a trend toward worse outcomes in patients with elevated CRP. The Kaplan–Meier survival analysis was performed to investigate the combined effect of HPR and CRP levels on MACE-free survival (Figure 5).

**Figure 6:**
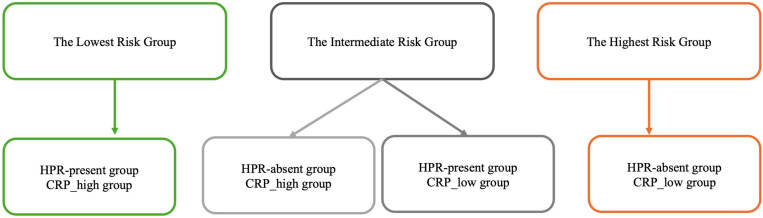
Risk stratification by CRP and HPR. HPR: high on-treatment platelet reactivity; CRP: C-reactive protein. The colors in Figure 6 are compatible with the colors of the curves in Figure 5.

Analysis of the survival curves showed the possibility of distinguishing groups that characterize the degree of risk, dividing patients into separate prognostic categories, as shown in Figure 6. Patients in “The highest risk group” have the highest probability of experiencing MACE. This suggests a synergistic negative effect in which the co-occurrence of platelet reactivity and inflammation results in a cumulative risk greater than the effect of either factor alone. The complete opposite is true in the case of the low-risk group; the relative stability of this curve suggests that the absence of both HPR and significant inflammation confers a protective status in the post-STEMI period.

### 3.6. High On-Treatment Platelet Reactivity and Lipid Profile

**Figure 7:**
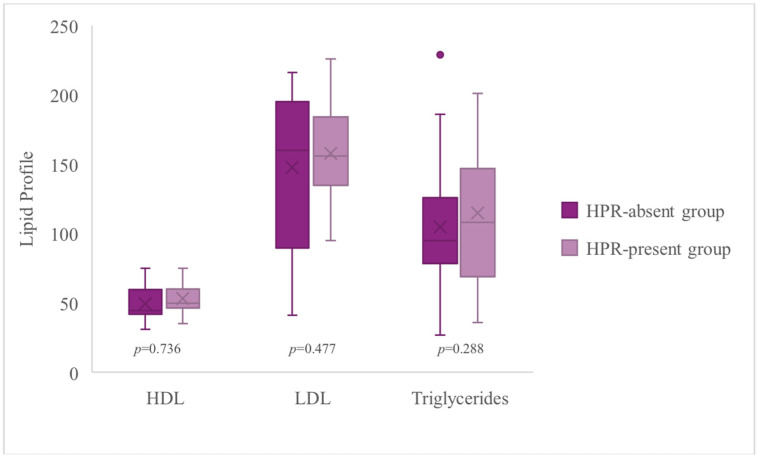
Comparison of lipid profiles (HDL, triglycerides, LDL) between HPR-present and HPR-absent patient groups. HDL: high-density lipoprotein; LDL: low-density lipoprotein; HPR: high on-treatment platelet reactivity; bar height: represents the mean value of the measured variable; “X” inside the bars: indicates the median value of the data; error bars: show the SD; whiskers: represent the minimum and maximum values; point above the whisker: represents an individual outlier. Poprawiłam p na italic na rysunku I wstawiłam nowy.

Figure 7 compares the high-density lipoprotein (HDL), cholesterol, triglyceride, and low-density lipoprotein (LDL) cholesterol levels between the HPR-present group and the HPR-absent group. The results indicate that patients with HPR tend to have a more atherogenic lipid profile. The median HDL level was unfavorably lower in the HPR-present group (48.50 mg/dL) compared to the HPR-absent group (59.50 mg/dL). However, the median triglyceride level was significantly higher in the HPR-present group (118.50 mg/dL) versus the HPR-absent group (73.50 mg/dL). These findings strongly suggest that HPR coexists with atherogenic dyslipidemia, characterized by low HDL and high triglycerides, which is a known risk factor for cardiovascular events.

**Figure 8:**
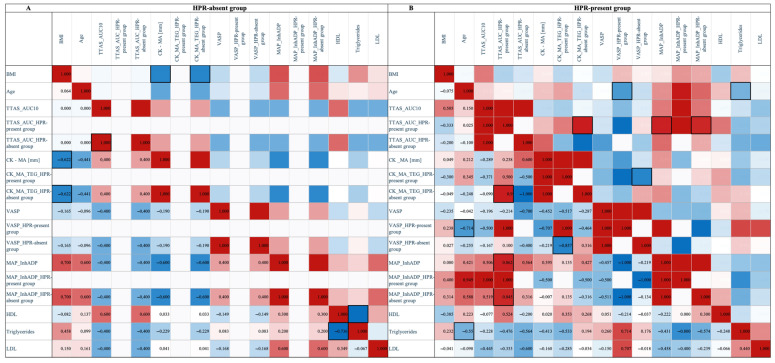
Spearman correlation matrix of HPR parameters and clinical variables in the HPR-absent group (**A**) and the HPR-present group (**B**); data shown in the thick box are statistically significant (*p* < 0.05). BMI: body mass index; TTAS: total thrombus formation analysis system; AUC10: area under the pressure–time curve for 10 min, PL chip for T-TAS; AUC: area under the curve; HPR: high on-treatment platelet reactivity; CK: kaolin test; MA: maximum amplitude; TEG: thromboelastography; VASP: vasodilator-stimulated phosphoprotein phosphorylation assay; MAP: platelet mapping; InhADP: percentage of inhibition of platelet mapping ADP; ADP: adenosine diphosphate; HDL: high-density lipoprotein; LDL: low-density lipoprotein.

Spearman’s correlation analysis, presented as two heatmaps (Figure 8), showed varying relationships between lipid profiles and platelet function parameters in STEMI patients, in the presence of HPR (HPR-absent group and HPR-present group). In the heatmaps, red indicates a strong positive correlation (coefficient near +1.0), while blue signifies a strong negative correlation (coefficient near −1.0). Intermediate colors and shades of white denote weaker associations. The analysis focused on the relationships between the lipid profile and various methods of assessing platelet function.

In the HPR-present group, the correlation analysis for HDL began with no significant association with the T-TAS AUC10 parameter. Subsequently, for the CK_MA_TEG method, the correlation became moderately positive. For the VASP parameter, the strength of the association significantly weakened, remaining a weak positive correlation. Finally, for the TEG MAP_InhADP method, the correlation shifted to weakly negative. A weak negative correlation was observed with the T-TAS AUC10 parameter for triglycerides. Transitioning to the CK_MA_TEG method, the correlation rapidly strengthened, becoming moderately negative. With VASP, a rapid increase in correlation strength and a shift to a firm positive direction were observed. In contrast, for the MAP_InhADP method, a rapid rise in the correlation strength and a change to a very strong negative direction were noted. For LDL values, a moderate negative correlation was observed with T-TAS AUC10. This correlation weakened for the CK_MA_TEG method, becoming a weak negative correlation, similar to VASP. Finally, as with triglycerides, for MAP_InhADP, the correlation changed direction and became very strongly negative. In contrast, in the HPR-absent group, the correlation profile is different, with much weaker correlations observed between the lipid profile and platelet function parameters.

## 4. Discussion

The selection of ticagrelor for this study was based on the prevailing dual antiplatelet therapy recommendations at the time of the study, specifically the updated ESC position statement on the use of DAPT in coronary artery disease 2017, prepared in collaboration with the European Society of Cardiology (ESC) and European Association for Cardiothoracic Surgery (EACTS) Working Group. For patients with ACS, regardless of the initial treatment strategy, ticagrelor (180 mg LD, then 90 mg twice daily) was recommended in combination with aspirin if there were no contraindications [12]. It should be noted that since the study’s inception, the guidelines have evolved. The guidelines for the management of patients with NSTEMI indicate that prasugrel should be preferred in this group of patients. This recommendation was influenced by the ISAR-REACT 5 study [13]. It compared two therapeutic strategies: prasugrel and ticagrelor. While both demonstrated superiority over clopidogrel, meta-analyses have suggested that ticagrelor may be superior in reducing mortality, whereas prasugrel offers greater protection against myocardial infarction. Furthermore, the choice of antiplatelet agents was influenced by availability; although cangrelor was approved in the US and EU in 2015, it was not accessible in Poland during the study period. The updated 2022 ESC guidelines, published by Kubica [14], clearly recommend cangrelor for treating ACS, but also note its continued unavailability in Poland. Further research is needed on treatment strategies with current recommendations for antiplatelet therapy in the context of HPR.

Although STEMI is traditionally considered a higher-risk condition than NSTEMI, the latter is often undertreated during follow-up [15]. Results from the PRAISE Registry revealed that NSTEMI patients present with a higher burden of comorbidities and cardiovascular risk factors, whereas STEMI patients are more often discharged on evidence-based pharmacological therapies. Despite these differences, 1-year clinical outcomes—including mortality, reinfarction, and major bleeding—were found to be largely comparable between the two groups [16]. These findings highlight the importance of optimal risk stratification and secondary prevention in NSTEMI patients. Another critical aspect in ACS patients is invasive and structural procedures, which may influence the platelet reactivity and short and long-term prognosis. Some studies present varying perspectives on platelet activity and coagulation depending on stent type. The type of stent deployed may influence coagulation, with drug-eluting stents (DES) generally considered more effective in reducing platelet activity than bare-metal stents. As confirmed in the review by Koźlik et al. [17], DES demonstrated lower restenosis rates due to the inhibition of cellular proliferation. Given that many STEMI patients are of advanced age and may have undergone prior cardiovascular procedures, their antithrombotic management is often heterogeneous [18]. Therefore, the tendency towards individualizing therapy in cardiological care is worth emphasizing.

In this prospective cohort study of patients with ACS/STEMI undergoing primary PCI, HPR predicted long-term adverse clinical outcomes. The results demonstrate that some STEMI patients exhibit HPR despite treatment with potent P2Y12 receptor inhibitors, such as ticagrelor. Some studies explained that presence of HPR may be related to the delay in ticagrelor absorption when morphine is administered [19,20]. A substudy of the ATLANTIC trial showed that morphine delayed the onset of platelet inhibition after a ticagrelor LD in patients with STEMI [21]. Similarly, the RAPID study [22] reported that while ticagrelor provides effective inhibition, its onset is delayed, with HPR maintained in 60% of patients, after two hours post-administration. Effective inhibition measured by VerifyNow occurs on average in 3–5 h after drug administration. However, as the platelet reactivity in the present study was assessed at 24 h, the effect of the simultaneous action of morphine is unlikely. Evidence also suggests that escalating the LD of ticagrelor above the standard 180 mg (to 270 or 360 mg) in patients with STEMI does not provide clinically significant benefits, as delayed drug absorption and active metabolite generation impair the pharmacodynamic response in the initial hours. This pharmacokinetic limitation cannot be overcome by dose modulation [23].

While the non-responsiveness to clopidogrel, often called resistance, is largely attributed to genetic factors [24], the response to ticagrelor is more complex. Ticagrelor has demonstrated efficacy in numerous patients with ACS, including those with clopidogrel resistance; however, there are still many reports of adverse reactions. Research indicates that while ticagrelor’s antiplatelet efficacy does not significantly differ across various genotypes, the risk of adverse effects, such as bleeding and dyspnea, shows substantial inter-individual variation [25,26,27]. It is noteworthy that studies on ticagrelor polymorphisms largely concern the Asian population.

The ONSET/OFFSET study by Gurbel et al. demonstrated that ticagrelor achieves a faster, more potent, and more predictable inhibition of platelet function compared with high-dose clopidogrel [28]. In the present study, the reference values of defining HPR were intentionally lower for VASP and TTAS, PRI > 30% instead of 50% [7], T-TAS_AUC10 > 50 AUC instead of 260 AUC [8], respectively. This adjustment reflects an emerging consensus that lower thresholds are necessary, particularly when comparing ticagrelor with clopidogrel. For instance, studies by many authors [9,10,11] have reported that mean platelet reactivity values in ticagrelor-treated patients are significantly lower, averaging approximately 20 AUC for TTAS and 10–16% for PRI. The lower threshold allowed us to identify a wider range of patients who did not respond adequately to the strength of the drug used. This also demonstrates current trends in cardiac care, where the potential of a more personalized approach to antiplatelet therapy is being explored.

Hemostasis is a dynamic process responsible for keeping a balance between coagulation and fibrinolysis, where both platelets and the coagulation system play a pivotal role in the pathogenesis of thrombosis [29]. The efficacy of P2Y12 inhibitors can be limited by HPR, which different biological assays can evaluate. This study used three independent methods to characterize the coagulation system and platelets in ACS/STEMI patients on DAPT with aspirin and ticagrelor. Although platelet function testing is a valuable tool in pharmacodynamic studies, only some institutions have protocols, and its clinical application remains limited. The HARMONIC trial [30] indicated that measurements from the VASP assay, the VerifyNow device, and the Multiplate analyzer show comparably strong negative correlations with ticagrelor and its active metabolite concentrations. Although still underestimated, platelet function testing is a tool that can help make better clinical decisions [31]. Our findings confirm that the detection of HPR varies depending on the assay used. This is probably partly related to the different mechanisms of action of these tests—VASP directly assesses P2Y12 receptor inhibition, and TTAS provides an overall insight into primary hemostasis. At the same time, TEG evaluates the entire process of in vitro clot formation.

Furthermore, platelet mapping by TEG assay provides a comprehensive view of the patient’s hemostasis to help assess risk and direct therapy [32]. It is essential because, in some clinical cases, the percentage of inhibition does not tell the complete information about bleeding and thrombosis risk. Patient response to antiplatelet therapy is variable; for example, up to 30% of patients on clopidogrel [33] achieve sub-therapeutic inhibition. Consequently, reducing platelet activity can still put them in a hypercoagulable or hypocoagulable state, depending on their baseline reactivity. In clinical practice, even in 10% of patients, despite the use of this antiplatelet therapy, recurrence of ischemia occurs as early as the first year after the ACS episode [34]. It has been confirmed that the treatment adherence is particularly impaired in the group of patients with diabetes [35,36]. This suggests the need for a more precise determination of the mechanisms leading to the modulation of the coagulation system, both at the level of drugs, comorbidities, and others.

Adherence is crucial in assessing the long-term effectiveness of therapeutic interventions. As defined by the World Health Organization, adherence is not only the extent to which the patient follows medical instructions but also a person’s behavior–following a diet and/or executing lifestyle changes. The level of adherence ≥ 80% is generally considered indispensable for the effectiveness of long-term therapy [37]. There are a lot of tools available for adherence measurements. The choice of the most suitable method to assess treatment adherence should be based on the specificity of the clinical setting. Methods for adherence evaluation are classified as subjective and objective or direct and indirect [38]. The patients who participated in our study were under the constant care of a cardiology clinic, and it is essential to emphasize that all received detailed education from medical staff upon discharge and at each follow-up visit. Education included a discussion of the importance of continuing antiplatelet therapy, the potential consequences of discontinuing it, and the impact of non-adherence on the risk of cardiovascular complications, including MACE. It is also important to note that according to the ESC guidelines for the management of patients with acute MI, therapy in these patients includes DAPT for 12 months, angiotensin-converting enzyme inhibitors (ACEI) or angiotensin receptor blockers if ACEI are contraindicated, beta-blockers, and statins [39]. A lack of studies in the literature directly addresses the adherence or extend beyond 12 months. While the PEGASUS-TIMI 54 [40] trial examined long-term ticagrelor therapy for up to three years, it focused on MACE reduction rather than adherence.

This analysis demonstrates that combining HPR and CRP status allows for a more detailed and clinically relevant risk stratification of STEMI patients. The most significant finding is that a prognostic hierarchy emerged among the four groups. While the individual markers of HPR and CRP showed trends toward worse outcomes, their combination uncovers a potential synergistic interplay between thrombosis and inflammation. A similar analysis was performed by Marcucci et al. [41], where the ROC curve analysis identified specific thresholds for LTA and CRP that were predictive of mortality. Elevated CRP is a well-established prognostic marker for MACE and mortality for cardiovascular reasons [42,43,44]. Although there is heterogeneity in the predictive value of CRP, the general consensus acknowledges a relation between CRP and cardiovascular disease.

The combination of low HDL and high triglycerides is a recognized pattern known as atherogenic dyslipidemia, which is a strong risk factor for the development and progression of atherosclerosis and cardiovascular events. The lower HDL level weakens the reverse cholesterol transport process, while elevated triglycerides contribute to forming small dense LDL (sdLDL) particles. These sdLDL particles more easily penetrate the arterial wall and undergo oxidation, initiating the atherosclerotic process. Moreover, inflammation can transform HDL into dysfunctional proinflammatory particles, especially in the case of LpA-I: AII particles; higher concentrations of these particles are associated with a more severe acute phase response during STEMI [45]. Consequently, HPR may function not only as an indicator of thrombotic risk but also as a marker for patients with advanced atherosclerosis, underscoring the necessity for aggressive and personalized management of lipid disorders in this population. These results emphasize the need for comprehensive evaluation and aggressive personalized treatment of lipid disorders, particularly atherogenic dyslipidemia, in patients identified with high platelet reactivity.

Despite many effective therapies, the long-term risk of re-hospitalization and mortality after STEMI remains high. Platelet reactivity monitoring at discharge may stratify patients at risk for out-of-hospital long-term clinical events like MACE. Current guidelines indicate a preference for using newer-generation P2Y12 inhibitors based on the TRITON and PLATO trials [46,47,48]. However, further investigation is needed to determine whether STEMI patients with HPR should benefit from routine monitoring and tailored therapeutic strategies to lower the risk of future ischemic events. 

## 5. Study Limitations

Our study had some significant limitations. First, there was a relatively small number in the study group and, consequently, limited statistical power and generalization of the findings. Second, the limited time points are 24 h after DAPT. There were no earlier time points; so, it may fail to detect early potential non-responsiveness. Furthermore, the observed correlations should be considered exploratory and hypothesis-generating and not definitive. We had patients with only one therapeutic strategy (DAPT-aspirin and ticagrelor). Finally, we did not assess the types of stents, the count and location of the infarct injury, or details of other invasive procedures.

## 6. Conclusions

The study confirms that patients with HPR have a higher risk of MACE events over a 5-year follow-up period. Furthermore, the coexistence of elevated CRP or lipid profiles is associated with a poorer long-term prognosis post-STEMI. These findings suggest that monitoring the CRP, lipid profile, and platelet reactivity are valuable components for comprehensive risk stratification in the management of STEMI patients.

A fundamental challenge persists in the standardized definition of HPR, as evidenced by the discordance among the different assessment methods utilized in this study, each identifying a distinct patient group. The specific recommendations regarding platelet reactivity are essential for selecting appropriate treatment and patient prognosis. Further research is needed, particularly on the increasingly frequently raised need to individualize antiplatelet therapy. This study may contribute to the development of standardized protocols for the routine assessment of platelet function regarding the benefits of long-term treatment.

## Figures and Tables

**Table 1:** Compatibility between HPR detection methods.

	TTAS_AUC10	CK_MA_TEG	VASP_PCI	MAP_InhADP
TTAS_AUC10	42.11	15.79	10.53	10.53
CK_MA_TEG	15.79	30.56	5.56	0.00
VASP_PCI	10.53	5.56	22.22	2.78
MAP_InhADP	10.53	0.00	2.78	11.11

Data are shown as a visual demonstration of the strength of concordance, ranging from darker pink for high values to light pink for low values (the darker the color, the higher the percentage of patients with HPR detected by both methods). T-TAS: total thrombus formation analysis system; AUC10: area under the pressure–time curve for 10 min, PL chip for T-TAS; CK: kaolin test; MA: maximum amplitude; TEG: thromboelastography; VASP: vasodilator-stimulated phosphoprotein phosphorylation assay; PCI: percutaneous coronary intervention; MAP: platelet mapping; InhADP: percentage of inhibition of platelet mapping ADP; ADP: adenosine diphosphate.

**Table 2:** Baseline characteristics of study participants.

Variable	Overall Study Population (*n* = 36)	HPR-Present Group (*n* = 22)	HPR-Absent Group (*n* = 14)	*p*-Value
**Age [years]**	62.00 (7)	62.00 (4)	63.50 (28)	0.435
Male *	66.7 (24)	54.5 (12)	85.7 (12)	0.053
**BMI**	27.64 (3.62)	27.84 (4.69)	26.75 (2.77)	0.935
Hyperlipidemia *	52.2 (12)	43.8 (7)	71.4 (5)	0.221
Current smoker *	76.9 (20)	83.3 (15)	62.5 (5)	0.245
**LDL [mg/dL]**	167.00 (58)	165.50 (53)	176.00 (93)	0.736
**Triglycerides [mg/dL]**	92.50 (78)	118.50 (101)	73.50 (54)	0.477
**HDL [mg/dL]**	49.50 (17)	48.50 (15)	59.50 (27)	0.288
**T-TAS AUC10**	29.70 (45.7)	38.30 (59.2)	25.10 (21.3)	0.193
**CK_MA**	72.40 (3.5)	72.50 (3.3)	69.70 (4)	<0.001
**VASP (PRI)**	15.78 (8.98)	17.13 (15.92)	12.71 (9.59)	0.063
**MAP_InhADP**	93.750 (25.9)	93.75 (28.30)	88.85 (23.9)	0.638

Data are shown as median (IQR) *p*-values from U M-W or *—number (%), *p*-values from chi-square test; HPR: high on-treatment platelet reactivity; BMI: body mass index [kg/m^2^]; LDL: low-density lipoprotein; HDL: high-density lipoprotein; T-TAS: total thrombus formation analysis system; AUC10: area under the pressure–time curve for 10 min, PL chip for T-TAS; CK: kaolin test; MA: maximum amplitude; VASP: vasodilator-stimulated phosphoprotein phosphorylation assay; PRI: area under the platelet reactivity index; MAP: platelet mapping; InhADP: percentage of inhibition platelet mapping ADP; ADP: adenosine diphosphate; n: number of patents.

## Data Availability

The data presented in this study are available on request from the corresponding author.
